# Examination of *Clock* and *Adcyap1* gene variation in a neotropical migratory passerine

**DOI:** 10.1371/journal.pone.0190859

**Published:** 2018-01-11

**Authors:** Andrea Contina, Eli S. Bridge, Jeremy D. Ross, J. Ryan Shipley, Jeffrey F. Kelly

**Affiliations:** 1 Oklahoma Biological Survey, University of Oklahoma, Norman, OK, United States of America; 2 Department of Ecology and Evolutionary Biology, Cornell University, Ithaca, NY, United States of America; 3 Department of Biology, University of Oklahoma, Norman, OK, United States of America; 4 Corix Plains Institute, University of Oklahoma, Norman, OK, United States of America; University of Iceland, ICELAND

## Abstract

Complex behavioral traits, such as those making up a migratory phenotype, are regulated by multiple environmental factors and multiple genes. We investigated possible relationships between microsatellite variation at two candidate genes implicated in the control of migratory behavior, *Clock* and *Adcyap1*, and several aspects of migratory life-history and evolutionary divergence in the Painted Bunting (*Passerina ciris*), a species that shows wide variation in migratory and molting strategies across a disjunct distribution. We focused on *Clock* and *Adcyap1* microsatellite variation across three Painted Bunting populations in Oklahoma, Louisiana, and North Carolina, and for the Oklahoma breeding population we used published migration tracking data on adult males to explore phenotypic variation in individual migratory behavior. We found no correlation between microsatellite allele size within either *Clock* and *Adcyap1* relative to the initiation or duration of fall migration in adult males breeding in Oklahoma. We also show the lack of significant correlations with aspects of the migratory phenotype for the Louisiana population. Our research highlights the limitations of studying microsatellite allelic mutations that are of undetermined functional influence relative to complex behavioral phenotypes.

## Introduction

A migratory life history must entail physiological and morphological adaptations that allow for sustained movement as well as behavioral adaptations that permit an animal to migrate successfully in the face of uncertain weather conditions, food resources, and mortality risk [1]. Yet, the genetic mechanisms that underlie these physiological and behavioral traits are largely unidentified [2]. Epistatic effects, variation in gene expression, and allelic mutations can drastically affect animal behavior [3, 4]. Investigating correlations between genes and behavioral phenotypes, wherein one focuses on differential gene expression or allele polymorphisms (i.e. short sequence repeats within exons) that are likely to relate to a particular phenotype, can provide insight into the evolution of migration and its molecular control [5–8]. By comparing variation in these genes to the phenotype/s of interest, we may obtain new insight into the underlying genetic mechanisms and their evolutionary implications.

Previous investigations attempted to identify gene variations associated with a particular trait in several taxa, including insects, fish, and birds [9–14] and a few studies aimed to detect allele polymorphisms associated with migratory phenotypes. From these studies, two prominent candidate genes emerged: *Clock* and *Adcyap1* [15–19]. Both genes are related to regulation of circadian rhythms in animals, with allele size polymorphisms appearing to predict migratory timing and propensity for migration in some avian species. The onset of migration behavior in most birds appears to be controlled by an endogenous clock that is modulated by photoperiod. The *Clock* gene is thought to influence the development of endogenous rhythms [20], as is *Adcyap1*, which controls the secretion of melatonin, a hormone that regulates sleep cycles [21].

Even though the molecular pathways of *Clock* and *Adcyap1* and their functions are relatively well known, the exact physiological linkage between allele variation at these genes and phenotype (e.g. behavior) is missing [22]. Nevertheless, some studies have shown that both genes showed allelic variation that correlated with migratory phenotypes [15, 23]. It therefore seems advantageous to further explore these possible linkages in different avian species, since these loci might be suitable predictors of migratory behaviors across species and even among divergent populations.

**Fig 1:**
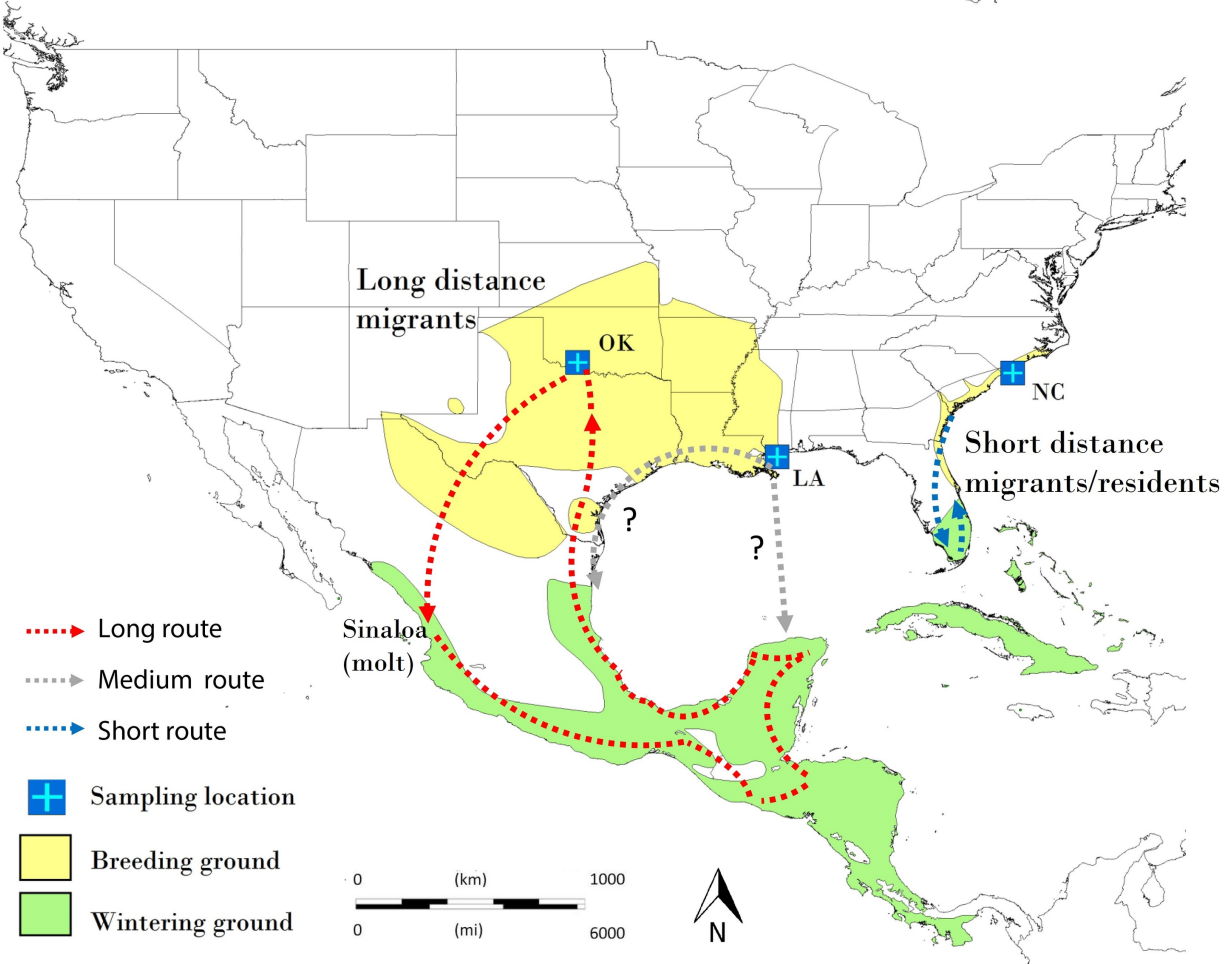
Painted Bunting distribution. The breeding and wintering ground are indicated in yellow and green, respectively. Blue crosses indicate sampling locations in Oklahoma (OK), Louisiana (LA) and North Carolina (NC). Dotted-line arrows and question marks indicate hypothetical migratory routes.

The Painted Bunting (*Passerina ciris*) is a Neotropical migratory songbird of small body mass (<20g) which breeds in two disjunct areas in western and eastern North America (Fig 1). These western and eastern populations show differences in their migratory and molt patterns and are recognized as two subspecies [24, 25]. At the end of the breeding season in late July the vast majority of birds in the western breeding population (*Passerina c*. *pallidior*) migrate from the southern Great Plains of the United States to northwestern Mexico to molt, and thereafter continue their migration to wintering grounds in southern Mexico and Central America [26, 27]. By contrast, individuals from the eastern population (*Passerina c*. *ciris*) breed along the Atlantic coast from North Carolina to central Florida and molt at the breeding grounds prior to commencing their fall migration [28]. The eastern breeding population shows a mix of residents and migrants, with only part of the population migrating to the wintering sites in southern Florida, the Bahamas, Cuba, and the Caribbean [28, 29]. Plumage coloration and wing morphology variations have also been documented between the western and eastern populations. Adult males breeding along the Atlantic coast exhibit darker red breast feathers and have shorter wings compared to males of the western population [30]. Interestingly, such “eastern” plumage coloration is also reported among birds approximately between 96° and 97°W longitude, which is contiguous with the western population range. Likewise, wing measurements showed a gradual decrease in length among individuals sampled increasingly westwards from eastern Texas, particularly between 93° and 95°W longitude [29–31].

In addition to these regional differences in Painted Bunting migratory behavior, timing of molt, and feather morphologies, it is also established that there is considerable variation in migratory behavior among individuals from a single western population. Painted Buntings from southwestern Oklahoma tracked with miniaturized light-level geolocation data loggers (hereafter “geolocators”) showed variation in the initiation and duration of fall migration [26]. The variation in migratory and molting behavior found at both inter- and intra-population levels makes the Painted Bunting an ideal candidate to investigate possible behavioral phenotype correlations to *Clock* and *Adcyap1* allele polymorphisms.

The present study seeks to establish or refute the validity of the genotype-phenotype correlations associated with microsatellite variations at *Clock* and *Adcyap1* in the Painted Bunting. To accomplish this goal we assessed *Clock* and *Adcyap1* allele variation in three geographically distant Painted Bunting populations. Beyond inter-population differences, we were also able to evaluate allelic variation at these genes relative to intra-population individual differences in migratory strategies, as revealed by light-level geolocators tracks [26]. Our assessment of expressed migratory behaviors relative to genetic markers is, therefore, based on observations in a natural setting, as opposed to lab-based observations.

## Materials and methods

### Sample collection

For *Clock* and *Adcyap1* analysis, we collected a total of 60 blood and feather samples from adult male Painted Buntings [32] trapped with mist-nets at three geographically distant breeding sites (Fig 1). The westernmost sampling site was the Wichita Mountains National Wildlife Refuge, Oklahoma (OK; N34.4 –W98.4), where we collected blood samples from 19 adult (after second year) males that were also equipped with geolocators [see 26 for complete methodology].

The next sampling site was Johnson Bayou, Louisiana (LA; N30.0 –W89.1), which is near the eastern extent of the larger western population. These birds represent, at least geographically and in some aspects of their morphology, an intermediate population between Oklahoma and the Atlantic coast. At this site, we obtained 21 primary or secondary feathers plucked from migrants arriving at the breeding grounds early in spring migration during the years 2011, 2012, and 2013. We measured body mass (g) and wing chord (mm) for each individual. In Louisiana, at a second field site (LA; N31.3 –W91.3), we also collected 16 blood samples which were used for neutral loci analysis (see next section).

We collected blood samples from 20 adult male Painted Buntings at Bald Head Island, North Carolina (NC; N33.5 –W78.0), which is a representative site of the allopatric eastern breeding population. In North Carolina, as in Oklahoma and in the second field site in Louisiana, we collected ~40 ul whole blood from the brachial vein of each individual and stored these samples in 0.4 ml of Queen’s lysis buffer at 4°C for later genetic analyses.

All work with animals was performed with relevant state and federal banding permits (Permit #23215) and was approved by the Institutional Animal Care and Use Committee of the University of Oklahoma (Protocol #R12-019).

### DNA extraction and genotyping

We carried out DNA isolation and purification from stored blood using DNeasy Blood and Tissue Kit (QIAGEN, Valencia, CA, USA). For feather DNA, we isolated DNA following a modified extraction and purification protocol that included an overnight tissue digestion step with no dithiothreitol [33]. We performed 10 ul (total volume) polymerase chain reaction (PCR) following [16] and using primers previously designed to amplify microsatellite repeat regions in avian species within *Clock* and *Adcyap1* genes [34]. We also genotyped four putative neutral microsatellite sequence repeats using primers specifically developed for Painted Buntings [35] (NCBI accession numbers: Pr032754276, Pr032754280, Pr032754287, Pr032754290).

We analyzed the PCR products on an ABI 3730 sequencer with an internal size standard (Genescan LIZ-600, Applied Biosystems, CA). We then visualized and scored the alleles by sequence length using Peak Scanner 1.0 (Applied Biosystems, CA).

### *Clock* and *Adcyap1* allelic polymorphism, population differentiation and genetic structure

We used the program ARLEQUIN v.3.5 [36] to test for deviation from Hardy-Weinberg Equilibrium (HWE) and Linkage Disequilibrium (LD) and estimated the p-values through a Markov chain Monte Carlo (MCMC) algorithm of one million iterations and significance level α < 0.05 [37, 38]. We estimated population differentiation at each gene, *Clock* and *Adcyap1*, and at the neutral loci through FST and RST statistics and significance level α < 0.05 [39] and calculated the genetic distances between all pairs of populations (δμ^2^) based on a microsatellite stepwise mutation model [40]. We then used δμ^2^ to build a tree diagram with the Neighbor-joining method to illustrate genetic distances at *Clock* and *Adcyap1* [40, 41].

We implemented a Bayesian population clustering algorithm in the program STRUCTURE [42] and analyzed *Clock* and *Adcyap1* separately to determine the contribution of each locus to the observed population differentiation. The parameters for our Bayesian population clustering model included assumptions of correlated allele frequencies, admixed populations, and incorporated the sample locations of origin (LOCPRIOR) to assist the clustering algorithm [43]. For each value of K ranging from 1 to 3, we ran 10 simulations with 10^6^ MCMC repetitions each, which included an initial burn-in of 10^5^ iterations. We then plotted the log probability [L(K)] of the data over multiple runs in STRUCTURE HARVESTER [44] to determine the most likely number of clusters among the samples [45]. We used the software CLUMPP to merge the models with the highest likelihood assignment values and used the software DISTRUCT to visualize the results [46, 47]. We tested for allele size differences across Oklahoma, Louisiana, and North Carolina populations by implementing unpaired Student’s t-tests.

We also implemented a spatially explicit clustering algorithm in the R program TESS3 [48] and computed ancestry coefficient estimations across populations based on *Clock*, *Adcyap1*, and four putative neutral genotypes. TESS3 implements least-squares and matrix factorization [49, 50] providing a population Q-matrix clustering output similar to STRUCTURE and therefore suited for result comparisons and cross-validations.

### Migratory phenotype-genotype associations

We examined the relationship between microsatellite allele size for each candidate gene (*Clock* and *Adcyap1*) and a set of phenotypic traits presumably under strong selection. We conducted our correlation analysis for the Oklahoma and Louisiana populations based upon linear regressions and with a significance level α < 0.05.

For the Oklahoma population we considered the following migratory phenotypic traits: (i) departure dates from the breeding grounds in southwest Oklahoma in late July and early August; (ii) arrival dates at the molting grounds in northwestern Mexico in late summer; (iii) duration of migration from the breeding to the molting grounds (S1 Table). The data and details used in this analysis were obtained from published geolocator tracks [26].

For the Louisiana population we considered the following phenotypic traits: (i) arrival dates at Johnson Bayou, (ii) body mass, and (iii) wing chord length (S1 Table) because body mass (i.e. accumulation of pre-migratory fat as energy reserve) and wing morphology have often been shown to be related to different migratory strategies [51]. Statistical analyses were computed in R and XLSTAT [52, 53].

## Results and discussion

### Polymorphism at Clock and Adcyap1 and genetic differentiation between populations

We genotyped specific DNA microsatellite variations within *Clock* and *Adcyap1* and four putative neutral genes across three Painted Bunting breeding populations in Oklahoma, Louisiana, and North Carolina, and no loci failed PCR amplification (Table 1, S2 Table).

**Table 1:** *Clock* and *Adcyap1* allelic variation across populations. Gene diversity, allelic richness, minimum and maximum allele size, allele average, and most common allele (also reported as a percentage) are presented for each population.

	CLOCK							ADCYAP1						
						
Population	Gene	Allele	Allele size	Allele size	Allele	most	most	Gene	Allele	Allele size	Allele size	Allele	most	most
(sampling site)	diversity	richness	min.	max.	average	common	common	diversity	richness	min.	max.	average	common	common
							%							%
**Oklahoma**	0.62	6	277	285	278.8	277	58%	0.75	7	165	177	168.9	169	39%
**Louisiana**	0.6	4.9	277	286	278.7	277	60%	0.73	4.9	165	173	169.1	169	40%
**North Carolina**	0.51	2	277	280	278.5	280	75%	0.64	3	167	171	169.3	169	90%

We found that none of the three populations considered in this study deviated from HWE at *Clock* (p > 0.05), whereas both populations in Louisiana and North Carolina deviated from HWE at *Adcyap1* (p = 0.019; p = 0.001; respectively; S3 Table). We did not detect evidence for genetic LD (p > 0.05; S4 Table). None of the pairwise R_ST_ values based on either *Clock* or *Adcyap1* allele frequencies were significantly different among populations whereas pairwise FST allele frequencies showed differentiation between Oklahoma and North Carolina and between Louisiana and North Carolina at *Clock* and between Oklahoma and North Carolina at *Adcyap1* (Table 2).

**Table 2:** FST and RST values. FST and RST values for population differentiation at *Clock*, *Adcyap1*, and neutral loci computed in ARLEQUIN.

		Clock			Adcyap1			Neutral loci	
	Population	**Oklahoma**	**Louisiana**	Population	**Oklahoma**	**Louisiana**	Population	**Oklahoma**	**Louisiana**
	**Oklahoma**			**Oklahoma**			**Oklahoma**		
FST	**Louisiana**	0.002		**Louisiana**	-0.014		**Louisiana**	0.066*	
	**North Carolina**	0.117*	0.065*	**North Carolina**	0.012*	-0.006	**North Carolina**	0.096*	0.076*
	Population	**Oklahoma**	**Louisiana**	Population	**Oklahoma**	**Louisiana**	Population	**Oklahoma**	**Louisiana**
	**Oklahoma**			**Oklahoma**			**Oklahoma**		
RST	**Louisiana**	-0.025		**Louisiana**	-0.022		**Louisiana**	0.032	
	**North Carolina**	-0.009	-0.018	**North Carolina**	-0.01	-0.02	**North Carolina**	0.078*	0.002

Statistically significant pairwise differences (p < 0.05) are marked with asterisks.

The neutral loci dataset returned significantly different values for all of the FST pairwise comparisons between populations and in one RST pairwise comparison between Oklahoma and North Carolina. The similarity of FST results observed in the *Clock* and *Adcyap1* genes relative to the neutral genes may be interesting to note. Perhaps the evolutionary pattern (i.e. mutation rate) of *Clock* and *Adcyap1* may not be that different from neutral. Although we might tend to focus on results that detect positive selection, neutral evolution of major genes might be an overlooked possibility [54].

The Neighbor-joining trees based upon genetic distances (δμ^2^) identified population relationships discordantly. The sister population of Louisiana was identified to be Oklahoma at the *Clock* gene versus North Carolina at the *Adcyap1* gene (S1 Fig).

### Population genetic structure

**Fig 2:**
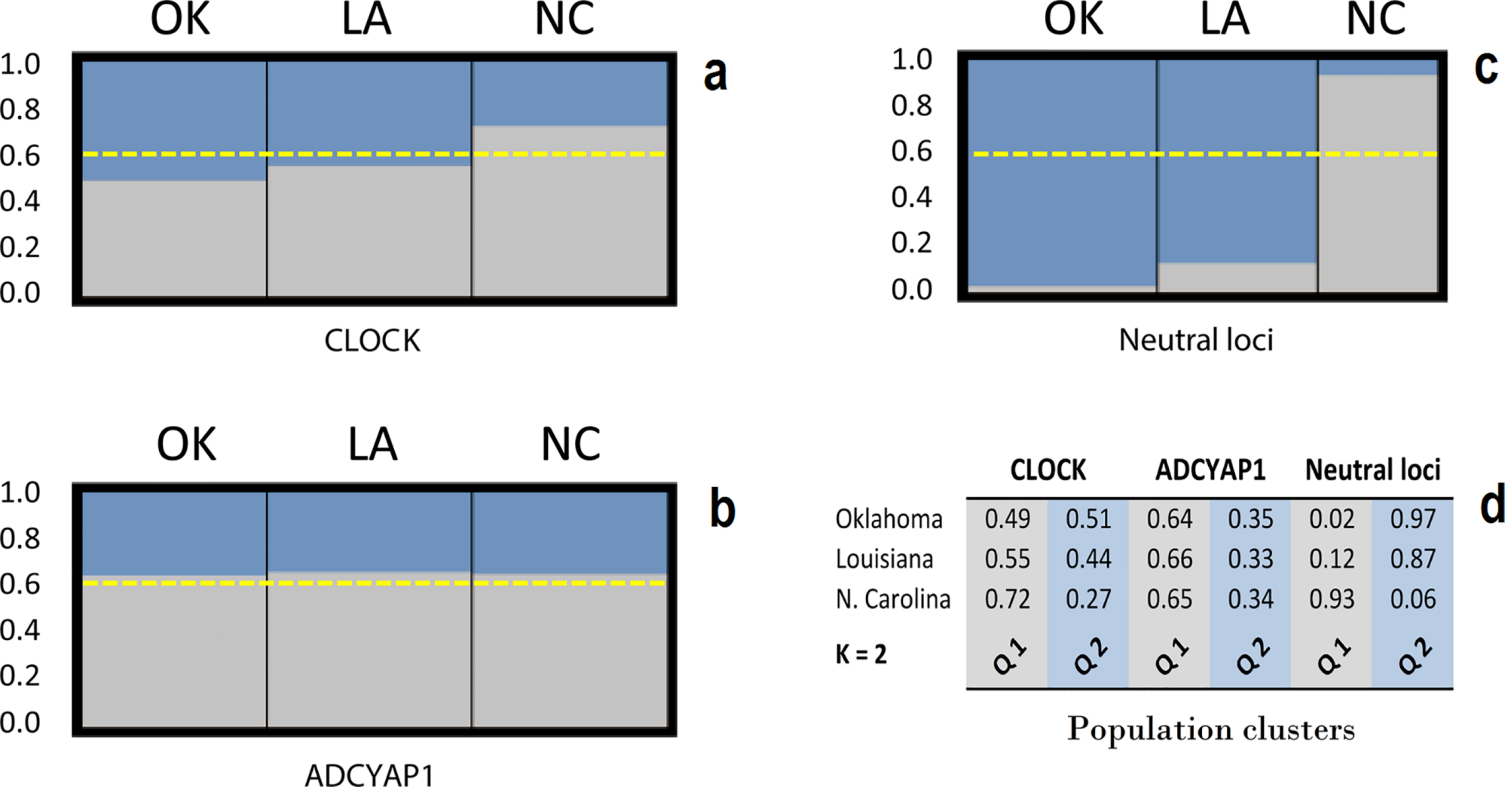
STRUCTURE Q matrix output for *Clock*, *Adcyap1*, and neutral loci. The graphs show the cluster assignment probabilities for Painted Buntings sampled in three populations (OK = Oklahoma; LA = Louisiana; NC = North Carolina) for K = 2. The probability (Q) of each population to be assigned to a cluster is shown on the vertical axes and in panel d. Each cluster is represented with a different color. The dashed yellow line represents the threshold probability (Q > 0.6) above which a population is considered part of a distinct cluster.

The Bayesian population clustering algorithm implemented in the program STRUCTURE found a small longitudinally-clinal differentiation from Oklahoma to Louisiana to North Carolina when cluster assignments were based on *Clock* genotypes, but indicated a complete lack of genetic structure based on *Adcyap1* perhaps because this locus is not in HWE (Fig 2; panel a-b). The clustering algorithm based on putative neutral loci showed a marked differentiation between the two western populations (Oklahoma and Louisiana) and the eastern population (North Carolina) (Fig 2; panel c-d). The most likely hierarchical group (K) was found to be equal to two when we implemented the ΔK method (*Clock* K = 2, mean LnP(K) = -153.8; SD = 1.72; *Adcyap1* K = 2, mean LnP(K) = -172.5; SD = 0.73; and neutral loci K = 2, mean LnP(K) = -756.8 SD = 7.53).

### Genotype-phenotype correlations at *Clock* and *Adcyap1*

Relative to all possible measures of migratory phenotype differences at the intra-population level (i.e., fall departure date, migration duration, and molt site arrival date among geolocator-tracked individuals in Oklahoma; spring arrival date, body mass, and wing chord among Louisiana individuals), we found no statistically significant correlation to microsatellite variation at *Clock* or *Adcyap1* genes and no differences between populations or mean allele size (Table 3 and Table 4).

**Table 3:** Correlation analysis between allelic and phenotypic variation. *Clock* and *Adcyap1* microsatellites did not correlate with any of the phenotypic variables investigated in Oklahoma and Louisiana buntings (p > 0.05). Allele-a indicates short alleles and allele-b indicates long alleles. Correlation values smaller than 0.01 are reported as nd (not detected).

	CLOCK		ADCYAP1	
Oklahoma	allele-a	allele-b	allele-a	allele-b
Departure dates (OK)	r = 0.24; p = 0.31	nd	r = 0.04; p = 0.86	r = 0.35; p = 0.13
Duration of migration	r = 0.01; p = 0.94	r = 0.11; p = 0.63	r = 0.11; p = 0.64	nd
Arrival dates (Sinaloa)	nd	r = 0.15; p = 0.52	nd	r = 0.09; p = 0.70
**Louisiana**				
Arrival dates (Johnson B.)	r = 0.17; p = 0.44	r = -0.02; p = 0.93	r = 0.16; p = 0.48	r = 0.11; p = 0.61
Body mass (g)	r = 0.08; p = 0.18	r = 0.09; p = 0.16	nd	r = 0.05; p = 0.32
Wing chord (mm)	r = 0.01; p = 0.59	r = 0.01; p = 0.85	r = 0.05; p = 0.32	r = 0.02; p = 0.51

**Table 4:** Differences in microsatellite allele size between populations at *Clock* and *Adcyap1*. No significant microsatellite allele size differences were found between Oklahoma, Louisiana, and North Carolina populations (p > 0.05). Allele-a indicates short alleles and allele-b indicates long alleles.

** **	** **	**CLOCK**		** **
population	allele-a	allele-b	allele-a	allele-b
	Oklahoma		Louisiana	
Louisiana	t = 0.31; p = 0.72	t = 0.03; p = 0.97		
North Carolina	t = -0.60; p = 0.54	t = 1.43; p = 0.16	t = -0.91; p = 0.36	t = 1.22; p = 0.22
** **	** **	**ADCYAP1**		** **
population	allele-a	allele-b	allele-a	allele-b
	Oklahoma		Louisiana	
Louisiana	t = -1.83; p = 0.07	t = 0.75; p = 0.45		
North Carolina	t = -1.81; p = 0.07	t = 0.04; p = 0.96	t = 0.33; p = 0.73	t = -0.92; p = 0.36

Neutral genetic markers have been extensively used to successfully distinguish individuals, populations, and species, and are relatively easy to isolate across the genomes of virtually all organisms [55]. Similarly, the ability to rapidly genotype candidate genes linked to short sequence repeats (microsatellites), which may be associated with functional control over certain phenotypic traits, could provide a powerful tool for studying the evolution of animal migration or other complex behavioral traits. In this study, we aimed to determine whether microsatellites within two candidate genes for migration, *Clock* and *Adcyap1*, are suitable candidates to inform the study of population-specific adaptive evolution in non-model organisms, especially pertaining to migration-driven divergence in songbirds breeding in North America. Our results, focusing on adult males, indicate that allele size polymorphisms do not significantly correlate with aspects of the migratory phenotype across three geographically distant Painted Bunting populations. The lack of such correlation in our study might be due to annual sampling differences and small sample size translating into low statistical power.

### Population structure revealed by *Clock* and *Adcyap1* microsatellite variation

The Bayesian population clustering analysis based on *Clock* and *Adcyap1* microsatellite allele frequencies showed marked differences compared to the analysis based on putative neutral loci. *Clock* gene analysis showed a gradual population differentiation that followed a longitudinal cline with samples from the North Carolina group being separated from the western and central groups (Oklahoma and Louisiana) while *Adcyap1* gene cluster results showed no differentiation across populations (Fig 2, panel a-b). By contrast, the neutral loci cluster analysis showed a sharp differentiation for birds sampled in North Carolina against birds sampled in Oklahoma and Louisiana (Fig 2, panel c). We are cautious with the interpretations of these results at least for two reasons: a) we do not know exactly where the microsatellite repeats are located in the Painted Bunting genome, and b) sample size limitations. The population clustering algorithm implemented in the R program TESS3 provided results that were generally in agreement with STRUCTURE even though the longitudinally-clinal differentiation from Oklahoma to Louisiana to North Carolina appeared accentuated for cluster assignments based on *Clock* genotypes, and evident for the Louisiana population based on *Adcyap1* (S2 Fig; panel a-b). In agreement with the STRUCTURE plot, TESS3 detected marked differentiation between the two western populations (Oklahoma and Louisiana) and the eastern population (North Carolina) based on putative neutral loci (S2 Fig; panel c-d).

We observe that STRUCTURE and TESS3 outcomes, overall, are in agreement with the population differentiation values reveled by FST and RST analyses, since both tests detected the highest signal of differentiation for computations based on neutral loci, and lower population differentiations for computations based on *Clock* and *Adcyap1* genes, respectively (Table 1). It is also worthwhile to note that FST values provide a measure of population differentiation under the assumption of an infinite allele model of mutation, whereas RST values measure genetic distance, which is calculated using a stepwise mutation model (SMM). Therefore, a lack of differentiation at *Clock* and *Adcyap1*, as indicated by RST values, might suggest incipient population differentiations, since computations of genetic distances based on SMM tests are generally less sensitive to this demographic scenario [56–58].

### Research of complex traits in non-model organisms and study conclusions

The genetic diversity and population structure of Neotropical migrants like the Painted Bunting are the direct consequence of several factors interacting with each other: migratory life-history, historical isolation, mutation, genetic drift, and natural selection. However, separating the contribution of each factor is a difficult task and the lack of clear knowledge of the mechanisms contributing to the observed genetic structure within a species represents a severe limitation in studies of population ecology and evolution.

*Clock* and *Adcyap1* might be important genes for the regulation of the migratory behavior in North American songbirds and other taxa. Nevertheless, our study shows that the specific microsatellite variations within *Clock* and *Adcyap1* might not be linked to observed migratory life-history variation in adult male Painted Buntings at neither inter- nor intra-population level.

Developing a cost-effective molecular approach that can explain behavioral variation is highly desirable and plausible knowing that even small allele-frequency differences across the genome (e.g. several loci), may determine drastic life-history changes [59]. However, our research highlights the limitations of studying allelic mutations that are of undetermined functional influence relative to complex behavioral traits. We recommend that forthcoming studies determine in what ways allele polymorphisms at candidate genes do or do not correlate with variation in migratory traits among species and populations.

Future studies on gene expression in non-model organisms will help to identify novel candidate genes and will also provide the opportunity to clarify whether our knowledge and assumptions on RNA transcriptions are correct in both model and non-model species [60]. Thus, we will likely need to look in depth into other candidate genes as soon as they become available [14, 61, 62]. Though the length and number of exons and the availability of detailed knowledge of phenotypic variation in non-model species may be limiting factors, the identification of phenotype-associated markers will accelerate the process of identifying variable genomic regions where candidate controller genes may reside [63].

## Supporting information

S1 Fig Genetic distances. The genetic distances (δμ^2^) computed for microsatellites within *Clock* and *Adcyap1* and visualized on a Neighbor-joining tree. Results based on *Clock* genetic distances indicated Louisiana (LA) and Oklahoma (OK) as sister populations. Results based on *Adcyap1* genetic distances indicated Louisiana and North Carolina (NC) as sister populations. Branches are not to scale.
(TIF)

S2 Fig TESS3 Q matrix output for *Clock*, *Adcyap1*, and neutral loci. The graphs show the cluster assignment probabilities for Painted Buntings sampled in three populations (OK = Oklahoma; LA = Louisiana; NC = North Carolina) for K = 2. The probability (Q) of each population to be assigned to a cluster is shown on the vertical axes in panel a, b, and c. Each cluster is represented with a different color. Panel d shows the sampling locations used to build the latitude/longitude matrix used in TESS3.
(JPG)

S1 Table Migratory phenotype of Painted Buntings. The top panel shows individual departure dates from Oklahoma at the end of the summer. The column “Migration” shows that the duration of fall migration, starting at the breeding ground in Oklahoma (USA) and ending at the molting ground in Sinaloa (Mexico), varies extensively between individuals. The details on the migratory phenotype reported in this table were obtained from published data (for illustrative purposes only; for details see Contina et al. 2013). *Clock* and *Adcyap1* microsatellite genotypes for each bird are reported in the right columns. The lower panel shows spring migration arrival dates, body mass, and wing chord for each individual sampled in Louisiana (Johnson Bayou). *Clock* and *Adcyap1* microsatellite genotypes for each bird are reported in the right columns.
(XLSX)

S2 Table Microsatellite allele size variation at *Clock* and *Adcyap1* across Painted Bunting populations. Values are reported for each allele (allele-A and allele-B) and for the average of the two alleles. Allele-A indicates short alleles and allele-B indicates long alleles.
(XLSX)

S3 Table Observed (Ho) and expected (He) heterozygosity at *Clock* and *Adcyap1*. Significant p values (p < 0.05) indicating deviation from Hardy-Weinberg equilibrium (HWE) are marked with asterisks.
(XLSX)

S4 Table Linkage Disequilibrium (LD) results for microsatellite at *Clock* (locus 1) and *Adcyap1* (locus 2). There is no indication that these two loci are in LD (p > 0.05).
(XLSX)
