# Supplementary figures and images for: Examination of Clock and Adcyap1 gene variation in a neotropical migratory passerine
Source: PLoS One. 2018 Jan 11;13(1):e0190859. doi: 10.1371/journal.pone.0190859 (PMC5764313; doi:10.1371/journal.pone.0190859)

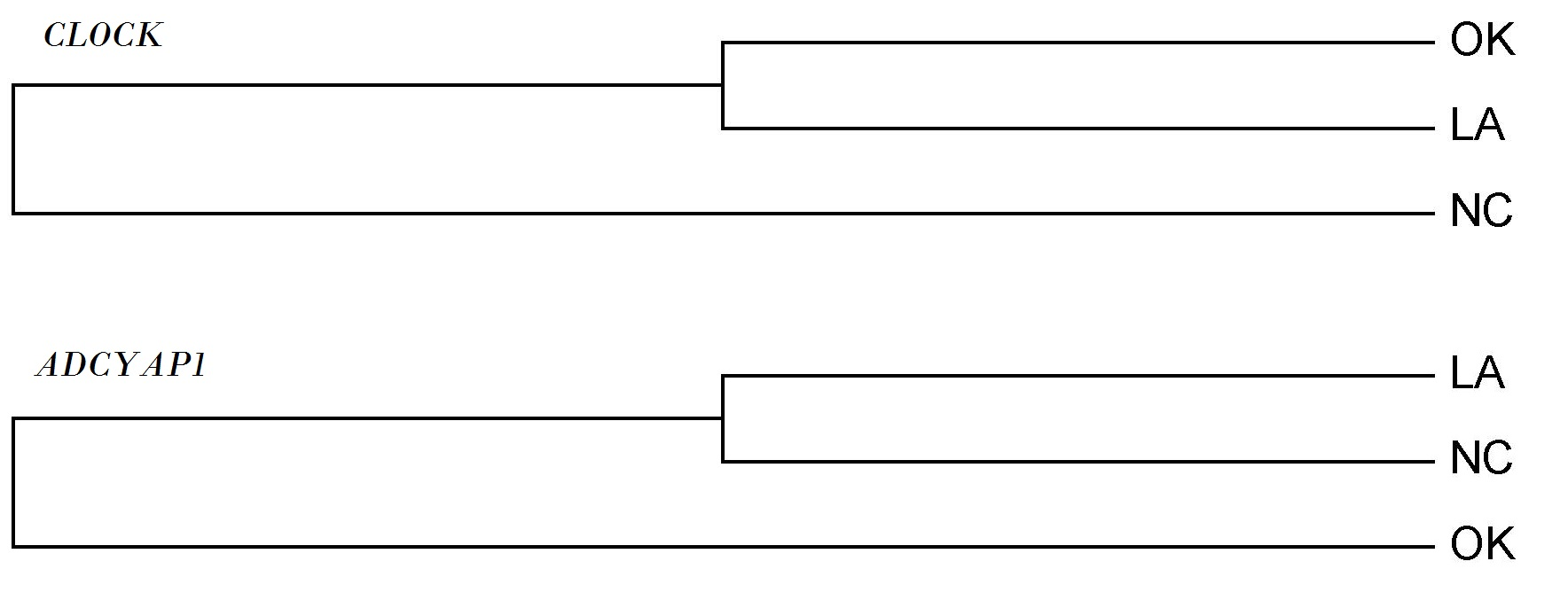

Supplement: S1 Fig — The genetic distances (δμ2) computed for microsatellites within Clock and Adcyap1 and visualized on a Neighbor-joining tree. Results based on Clock genetic distances indicated Louisiana (LA) and Oklahoma (OK) as sister populations. Results based on Adcyap1 genetic distances indicated Louisiana and North Carolina (NC) as sister populations. Branches are not to scale. (TIF) [file pone.0190859.s001.tif]

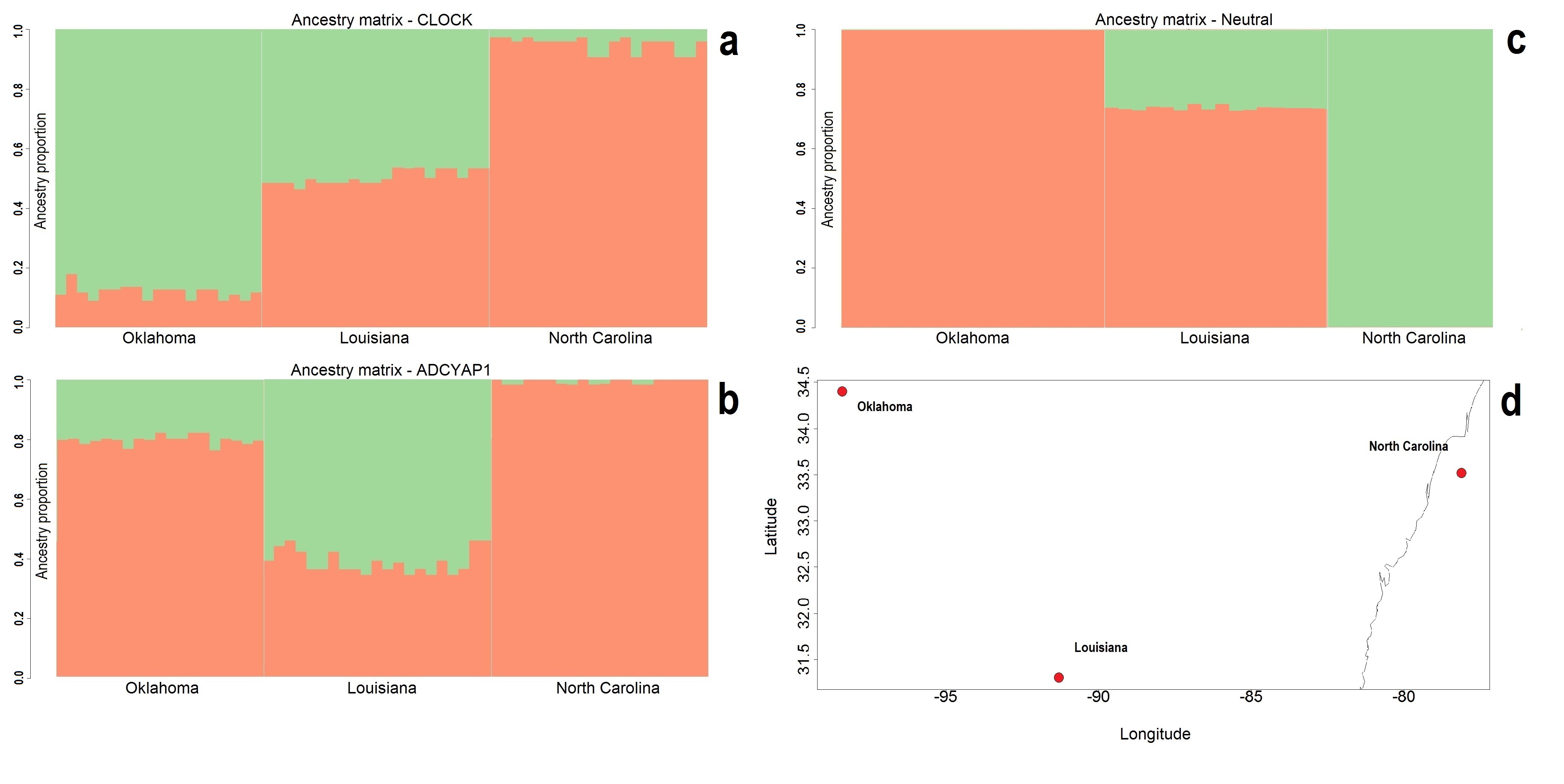

Supplement: S2 Fig — The graphs show the cluster assignment probabilities for Painted Buntings sampled in three populations (OK = Oklahoma; LA = Louisiana; NC = North Carolina) for K = 2. The probability (Q) of each population to be assigned to a cluster is shown on the vertical axes in panel a, b, and c. Each cluster is represented with a different color. Panel d shows the sampling locations used to build the latitude/longitude matrix used in TESS3. (JPG) [file pone.0190859.s002.jpg]
